# Vessel Labeling in Combined Confocal Scanning Laser Ophthalmoscopy and Optical Coherence Tomography Images: Criteria for Blood Vessel Discrimination

**DOI:** 10.1371/journal.pone.0102034

**Published:** 2014-09-09

**Authors:** Jeremias Motte, Florian Alten, Carina Ewering, Nani Osada, Ella M. Kadas, Alexander U. Brandt, Timm Oberwahrenbrock, Christoph R. Clemens, Nicole Eter, Friedemann Paul, Martin Marziniak

**Affiliations:** 1 Department of Neurology, University of Muenster Medical Center, Muenster, Germany; 2 Department of Ophthalmology, University of Muenster Medical Center, Muenster, Germany; 3 NeuroCure Clinical Research Center and Experimental and Clinical Research Center, Charité University Medicine Berlin and Max Delbrück Center for Molecular Medicine, Berlin, Germany; 4 Department of Neurology, Charité University Medicine Berlin, Berlin, Germany; 5 Department of Neurology, Isar-Amper-Klinikum, Haar, Germany; 6 Institution of Medical Informatics, University of Muenster, Muenster, Germany; Justus-Liebig-University Giessen, Germany

## Abstract

**Introduction:**

The diagnostic potential of optical coherence tomography (OCT) in neurological diseases is intensively discussed. Besides the sectional view of the retina, modern OCT scanners produce a simultaneous top-view confocal scanning laser ophthalmoscopy (cSLO) image including the option to evaluate retinal vessels. A correct discrimination between arteries and veins (labeling) is vital for detecting vascular differences between healthy subjects and patients. Up to now, criteria for labeling (cSLO) images generated by OCT scanners do not exist.

**Objective:**

This study reviewed labeling criteria originally developed for color fundus photography (CFP) images.

**Methods:**

The criteria were modified to reflect the cSLO technique, followed by development of a protocol for labeling blood vessels. These criteria were based on main aspects such as central light reflex, brightness, and vessel thickness, as well as on some additional criteria such as vascular crossing patterns and the context of the vessel tree.

**Results and Conclusion:**

They demonstrated excellent inter-rater agreement and validity, which seems to indicate that labeling of images might no longer require more than one rater. This algorithm extends the diagnostic possibilities offered by OCT investigations.

## Introduction

Optical coherence tomography (OCT) has come to be used increasingly to evaluate retinal degenerative changes involved in neurological diseases. Developed in the 1980s [1], today's modern spectral domain (SD) OCT scanners produce detailed cross-sectional and 3D-images of the eye. Thinning of the retinal nerve fiber layer (RNFL) measured by OCT has been widely described in patients with multiple sclerosis (MS) and MS-related optic neuritis [2]–[5]. Furthermore, some other neurodegenerative diseases, such as dementia, spinocerebellar ataxia or Parkinson's disease, were found to be associated with reduced thickness of the RNFL in SD-OCT scans [6]–[8], while others, such as amyotrophic lateral sclerosis, were not [9].

It is under discussion whether OCT has the potential to become a noninvasive, reproducible test for assessing axonal degeneration and whether it might be used as a valuable tool for measuring the therapeutic efficacy of potential neuroprotective agents [10]. This suggestion is based on the observation that retinal and cerebral atrophy are correlated [11]–[13].

In addition to RNFL thickness parameters, modern SD-OCT scanners provide additional information like confocal scanning laser ophthalmoscopy (cSLO) with infrared (IR) imaging. Additionally, the development of the eye tracker, which allows simultaneous investigation of the eye with two laser beams, ensures less eye-motion artifacts and highly comparable longitudinal examinations with reduced error rates.

Developments such as the ones outlined above enable us to collect a large number of data in a single examination, thus opening the door for multimodal examination of many neurological diseases.

Combining different technical approaches in a single investigation has numerous advantages:

From the viewpoint of patients: combined confocal scanning laser ophthalmoscopy and optical coherence tomography is a non-radioactive examination that can be performed in less than 20 minutes. In this context, papillary dilation is no longer required to ensure high-quality results. The burden of OCT investigations on the patient is low, resulting in a high acceptance rate for longitudinal investigations.
From the economic viewpoint: Compared to MRI scans, this approach can be handled with much less costly equipment and staff while also being faster.
From the viewpoint of research, in particular: multi-modality imaging opens the research spectrum and links different views on a particular disease.

Recently, consensus criteria for retinal OCT quality assessment (OSCAR-IB) have been published to increase the comparability and improve the quality management of OCT-images [14].

The main parameter analyzed in most studies is RNFL thickness, whereas lesser attention is paid to the retinal blood vessels. cSLO IR-imaging, however, is always combined with a OCT scan, which facilitates collection of additional information not only in patients with vasculopathy.

So far, there are no reliable and valid criteria for labeling blood vessels in cSLO IR-images recorded by OCT-scanners. Up to now, studies which describe labeling of retinal blood vessels refer to classical color fundus photography (CFP) images only.

A review of the ophthalmological literature yielded criteria originally developed for automatic analysis of CFP [15], [16]. As a result of their shared embryology, cerebral and retinal blood vessels share similar anatomical and physiological properties. In the recent past, the eye, the “window to the brain”, was used to investigate different neurological diseases. Changes in retinal vessels were detected in the context of many neurological diseases such as Alzheimer's disease or neuromyelitis optica [17], [18]. Especially for Alzheimer's disease, retinal vascular image analysis was described as a potential screening tool. These examples demonstrate the huge potential of retinal blood vessels examination for detecting preclinical diseases and for evaluating clinical courses.

The aim of this study was:

1. to develop reliable and valid criteria for labeling retinal blood vessels in cSLO IR-images,
2. to investigate whether the criteria defined for automatic analysis of CFP can also be applied for cSLO IR-images,
3. to propose standard operating procedures for further studies in neurological diseases.

The study was comprised of three portions:

(1) an exploratory part, (2) an adaptation of the criteria based on the initial results, and (3) a validation study comparing the results with CFP.

## Exploratory Study

### Subjects

273 blood vessels in both (14) eyes of 7 healthy volunteers (6 males, mean age 50.9±13.9 years) were labeled by two independent raters (CE, JM).

All subjects had normal visual acuity (20/20), normal visual fields and no ocular, metabolic or neurological diseases. The whole study was conducted according to the principles expressed in the Declaration of Helsinki. The institutional review board of the ethics committee of the University of Berlin, Charite, approved the study with volunteers. Participants provide their written informed consent on a standardized informed consent form approved by ethics committee.

### Methods

Retinal images were obtained using combined cSLO and SD-OCT imaging (Spectralis, Heidelberg Engineering, Software: Heidelberg EyeExplorer version 1.7.0.0) with the eye tracking function enabled.

Using automated eye tracking and image alignment based on cSLO images, the integrated software can be used to average a variable number of single images in real time (Automatic Real Time [ART] Module; Heidelberg Engineering), which significantly improves image quality. Furthermore, this technique ensures reliable follow-up measurements, as scans are recorded at exactly the same position as the baseline scan.

The IR-images were pseudonymized, exported by Heidelberg EyeExplorer, and uploaded to an ImagJ-Plugin for measuring vessel diameter (http://neurodial.de). Afterwards, blood vessels were labeled in cSLO images.

The inter-rater agreement between the two raters was measured using Cohen's kappa coefficient. According to Landis and Koch, strength of agreement was rated as poor (<0.00), slight (0.00–0.20), fair (0.21–0.40), moderate (0.41–0.60), substantial (0.61–0.80), or almost perfect (0.81–1.00) [19], [20].

### Test criteria

Criteria formerly reported in the literature, which had been developed for an automatic analysis of fundus images, were reviewed and eight criteria were selected for the exploratory analysis of the vessels and weighted equally to each other:

1. The central light reflex is wider in arteries and smaller in veins [15], [16].
2. Arteries are brighter than veins [15], veins appear darker and deeper than arteries [16].
3. Arteries and veins alternate near the optic disc [15], [16].
4. Arteries are 30% thinner than neighboring veins [15].
5. Arteries never cross arteries and veins never cross veins [16].
6. The angle between crossing vessels is almost 90°, and angles between outgoing vessels are between 30° and 45° [16].
7. Vessels should be seen in the context of the vessel tree [15].
8. Arteries take a straighter course than veins [16].

The raters labeled vessels with “A” for artery, “V” for vein and “U” for unknown.

### Results

The inter-rater agreement of the exploratory study showed a kappa of 0.602. The two raters marked 20.5% or 38.8% of 273 vessels as unknown. The disagreement-rate was 27.5%.

### Interpretation

The exploratory study showed a high rate of unknown vessels and substantial inter-rater agreement (κ =  0.602). There was a strikingly high difference between the raters for vessels labeled “unknown”.

**Figure 1:**
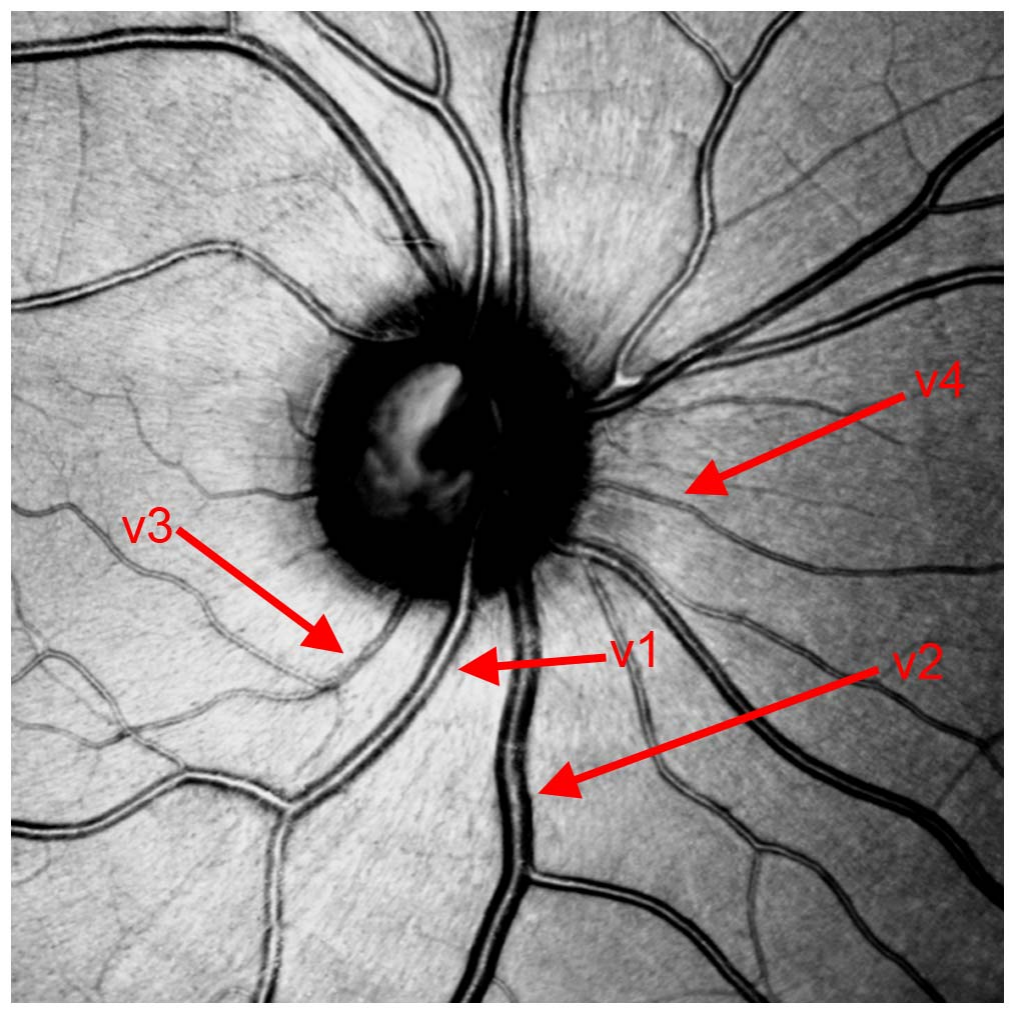
Vessels 1 (v1) and 2 (v2) are larger than vessel 3 (v3) and 4 (v4). V1 is an artery, v2 is a vein, v3 and v4 cannot be allocated clearly.

On the one hand, the main branches and bigger vessels were clearly labeled and the labeling left no space for interpretation. On the other hand, there are many vessels which leave some leeway for interpretation. The smaller the vessels were, the harder clear labeling became (figure 1).

The results of this pre-test demonstrated the need to rate the criteria and to modify them. Originally, the criteria had been established for an automatic analysis of CFP images. Consequently, they needed to be adapted to the nature of cSLO IR-images. The images generated by OCT scanners are black and white pictures, and the relevant criteria did not seem to be transferable on a 1∶1 basis.

### Test Modification

In view of the above, our next step was to develop an algorithm for labeling blood vessels which could not be identified clearly. A non-hierarchical application of the criteria in the exploratory study was followed by a weighting of the criteria in a consensus meeting which included all authors from Münster. Decisions were based on the initial results, on anatomical and physiological facts, and on the different technical features of the OCT and cSLO IR-Images.

### Main criteria

We defined two categories of criteria: main and additional criteria. Main criteria were based on anatomical or physiological correlates.

**Figure 2:**
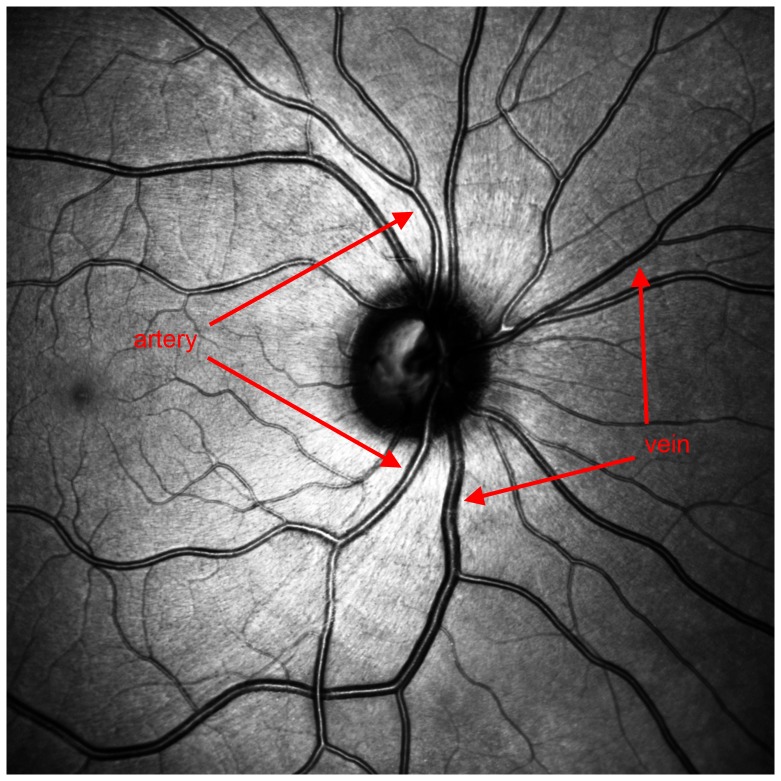
The artery shows a reflection-zone extending from the optic disc to the periphery of the retina. The reflection of the vein cannot be traced to the periphery. Compared to the venous cross section, the reflection is smaller. The venous vessel wall appears thicker.

**Figure 3:**
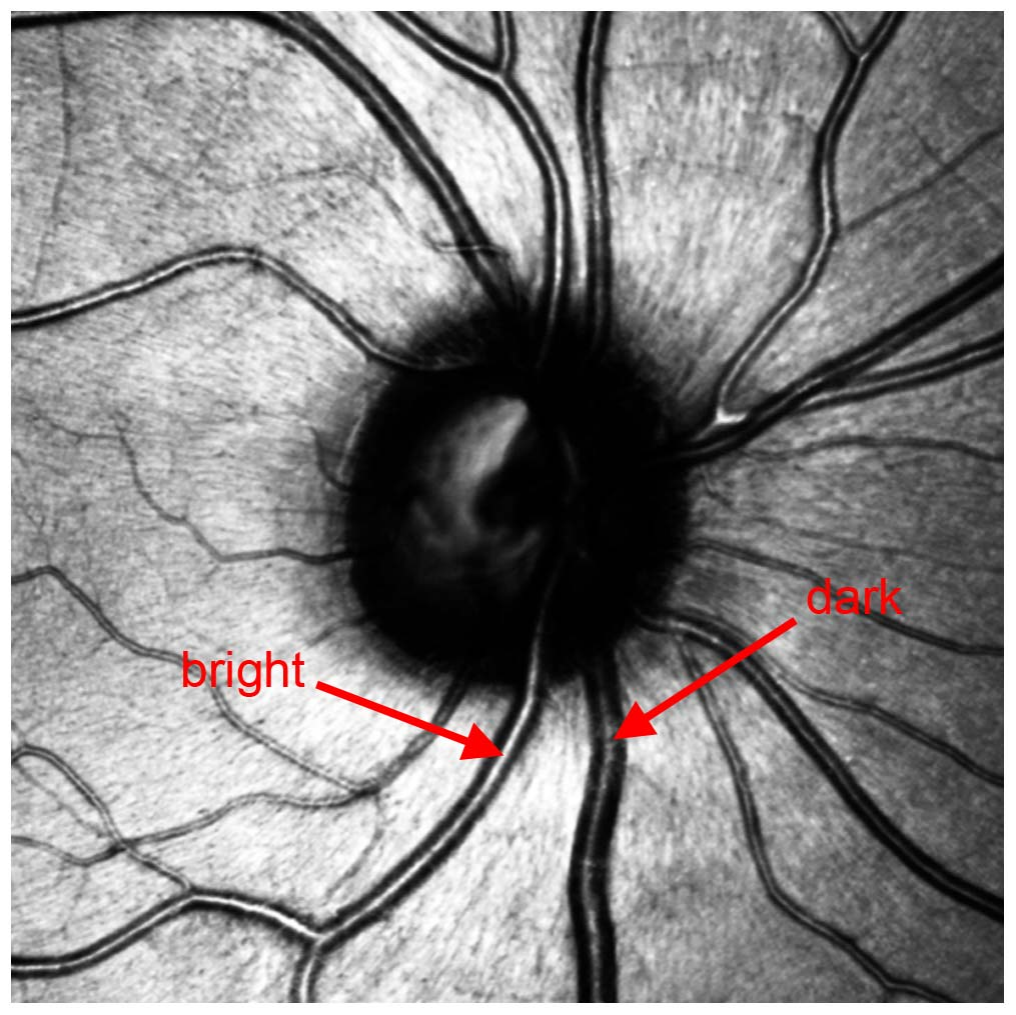
The darker vessel is a vein, the brighter an artery.

**Figure 4:**
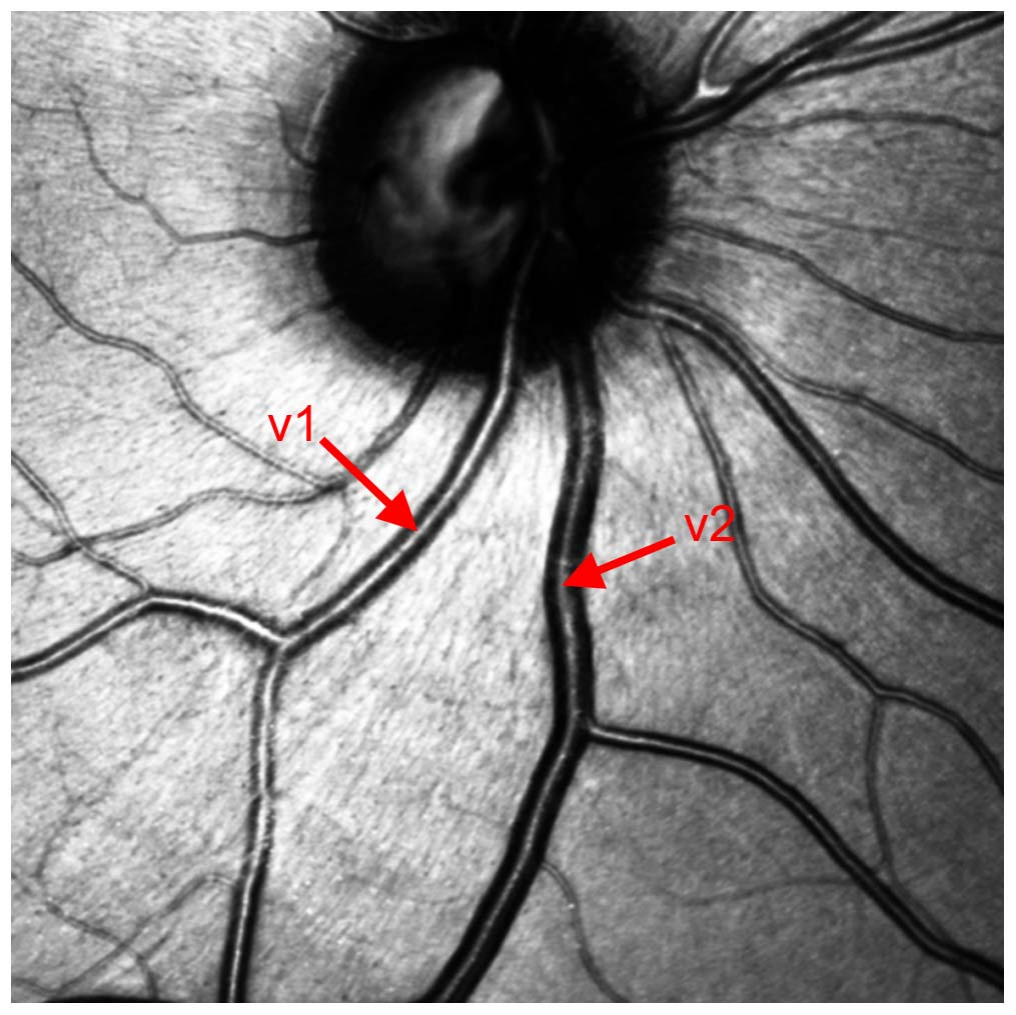
An example of the difference in size can be observed between vessel 1 and vessel 2. V1 is an artery, v2 is a vein.

**Figure 5:**
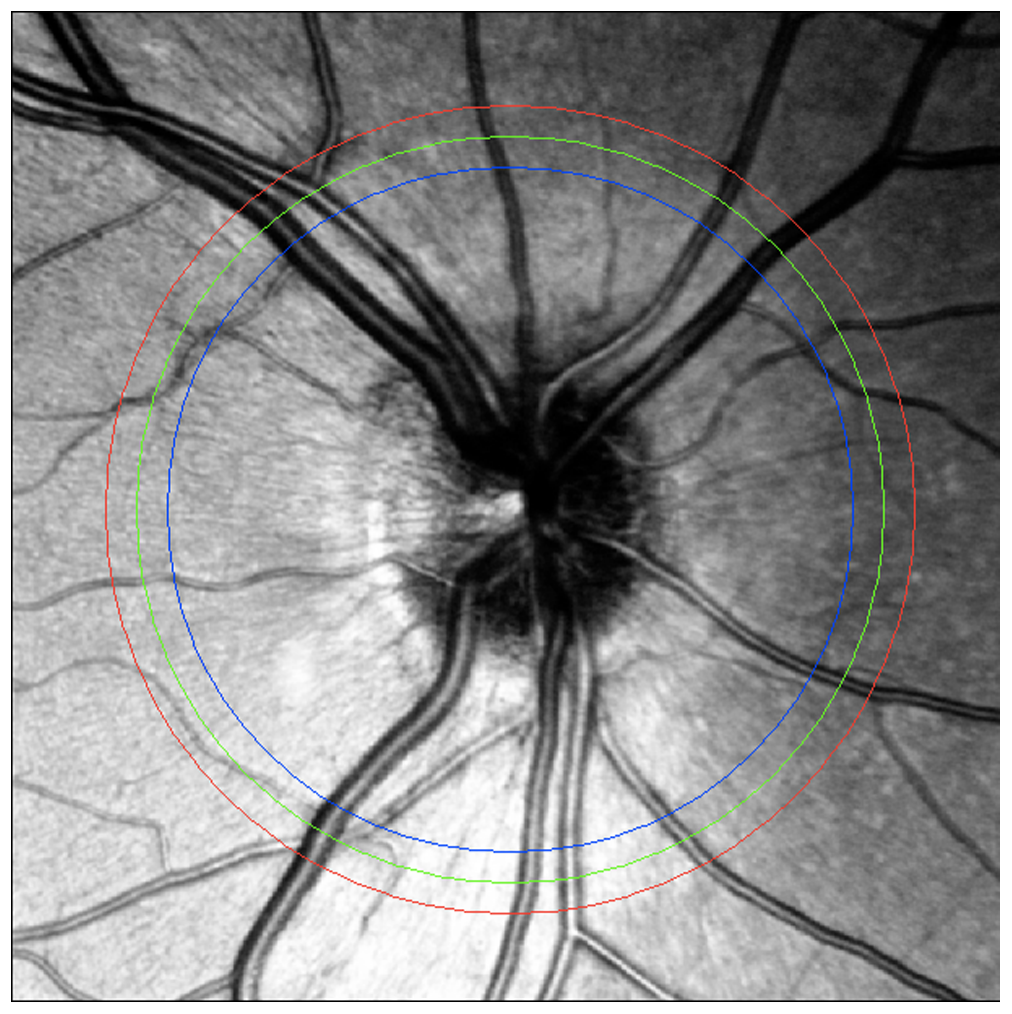
Three rings around the optic disc ensure vessel grading at consistent eccentricity for each vessel.

1. “The central light reflex is wider in arteries and smaller in veins.”
  Originally, this criterion was based on fundus images produced via the red channel mode, a special mode with colored filters in which veins show larger vessel edges and bigger color differences between the edge and the reflection zone in the middle of the vessel. In contrast, arteries appear lighter than veins (figure 2) [15].
  The reason for the varying size of the light reflex is the difference of the vessel walls of arteries and veins. The central light reflex (CLR), which is caused by light reflection from vessel surfaces, is a phenomenon which was first observed in images produced by light of a 600 nm wavelength [21], [22], but is also seen in 820 nm cSLO IR-images.
  Moreover, arteries have solid walls built by the tunica media, the middle layer of an artery wall. They are rich in muscle fibers and reveal more reflection compared to the vessel wall of veins. Veins show loosely packed vessel walls and the three layers of the wall, tunica intima, tunica media and tunica externa, merge with each other [23].
2. “Arteries are brighter than veins.”
  The brightness of arteries is also caused by the oxygen-enriched blood transported by them [15], [21]. This effect also shows in IR images [21].
  In contrast, the lumen of veins appears darker due to the circulation of deoxygenated blood (figure 3). In contrast to the first criterion, this criterion describes the brightness of the reflex rather than of its expansion (wider versus smaller).
3. “Arteries are up to 30% thinner than veins.”
  Because of the lower blood pressure, veins have bigger cross sections than their corresponding arteries. This is explained by the Hagen-Poiseuille equation (ΔP = 8µLQ/πr^4^). The volumetric flow rate (Q), the length of the pipe (L) and the dynamic viscosity (μ) do not change, which means that the cross section (r) will increase along with a decreasing blood pressure (figure 4).
  In this context, it should be noted that the main criteria apply for vessels on the same level only and that comparing vessels in the periphery of a 30° image with vessels in the center close to the optic disc, or vessels leaving the upper half of the optic disc with vessels in the lower half is not acceptable in view of the fact that the vessels change their morphology in their course (becoming smaller or bigger) and because the illumination differs depending on the various parts of the image. In the cSLO image, three rings around the optic disc ensure consistent eccentricity for each vessel when grading it (figure 5). Furthermore, the three rings were created to be used by an upcoming automatic vessel analyzing software.

### Additional criteria

Additional criteria are based on the experience of ophthalmologists and the raters' assessment. Their anatomical or physiological correlations are not as clear as for the main criteria.

**Figure 6:**
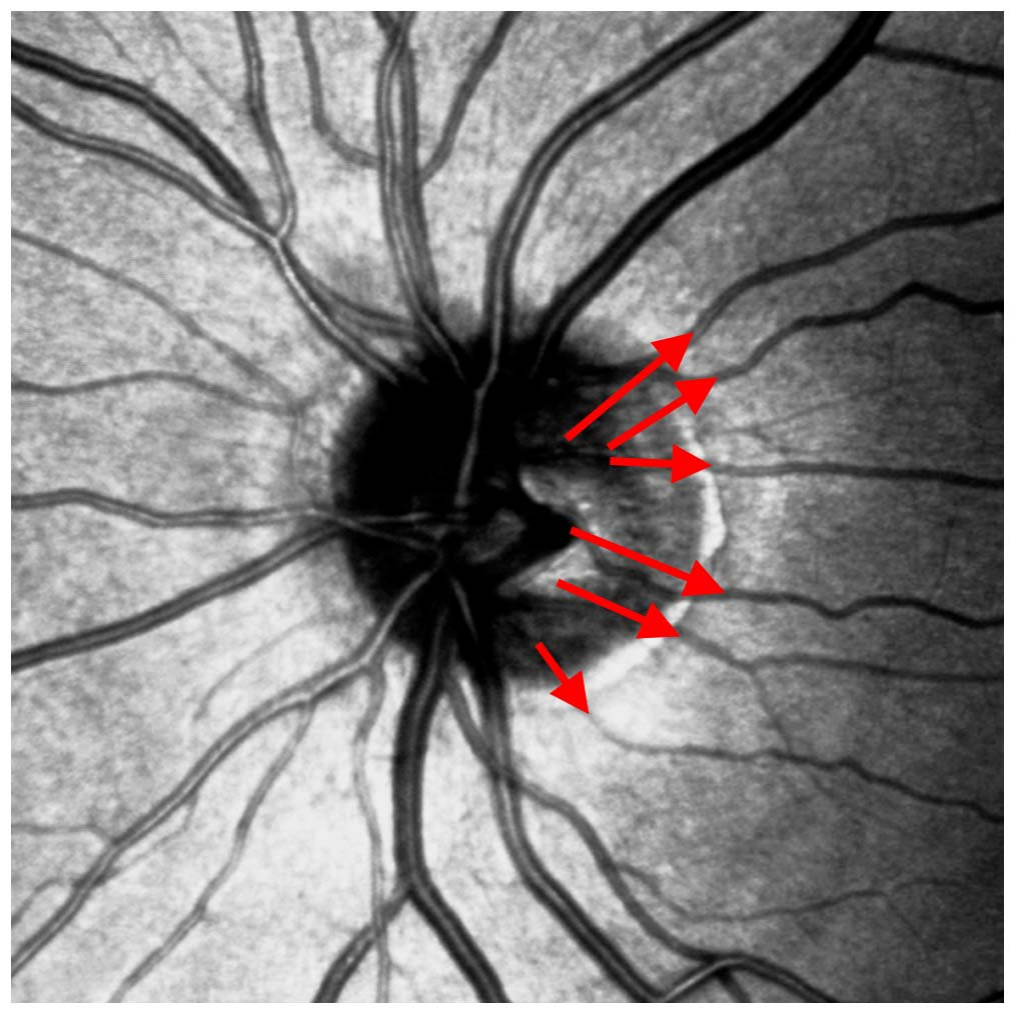
Alternate vessels with low variance of thickness and brightness.

1. “Arteries and veins alternate near the optic disc.”
  Near the optic disc, an artery runs next to a vein and vice versa. This means that an artery is surrounded by two veins and that a vein is surrounded by two arteries. It seems to be a very efficient differentiation criterion, because one labeled vessel is enough to specify the neighboring vessels.
  The blood vessels in the periphery, however, do not strictly follow this rule—it only applies before blood vessels begin to branch out. In cSLO IR-images, the center of the optic disc is often outshined. Consequently, the blood vessels are indistinguishable. Furthermore, very small blood vessels do not show the typical CLR or variance of thickness and brightness (figure 6). Using this criterion alone could therefore lead to errors comparable to those produced by a frameshift mutation: all subsequent vessels would be labeled incorrectly.
2. “Arteries never cross arteries and veins never cross veins.”
  This criterion underlines that if two blood vessels cross each other, the darker one must be the vein and the lighter one the artery [16].
3. “Vessels should be seen in the context of the vessel tree.”
  The idea of this criterion is to follow the course of vessels and find branchings. If a vessel can be labeled before the branching, it helps to determine the vessel parts after the branching; therefore unequivocal labeling of the part before the branching is absolutely necessary.
4. “Arteries take a straighter course than veins.”
  This observation is frequently cited and plausible in view of the physiological function of the arteries and veins. Veins drain the blood from wide tissue areas and a winding course will support this function.
  For the rater, the aforestated rule leaves room for interpretation and therefore does not seem very reliable. Moreover, blood vessels near the optic disc, whether veins or arteries, generally tend to have a straight course. This is why the difference in straightness between arteries and veins might not be very pronounced in this region.
5. “The angles between crossing vessels are almost 90°, whereas the angles between outgoing vessels range between 30° and 45°.”
  Although this rule is found in the literature [16] and although examples for this case could be observed, our exploratory study often yielded deviation from this rule.

Based on the results of our exploratory study and the considerations indicated above, we reassessed the criteria in an attempt to answer the following questions:

1. Are the main criteria correct? How much of them are required for an unequivocal identification of a blood vessel?
2. How are the additional criteria to manage?
  - Are additional criteria helpful if the main criteria are not adequate?
  - Which additional criteria are correct and which are not?
  - How many of the additional criteria are required for correct labeling without using the main criteria?
3. Is the validity of the test impacted by modifying the test criteria?

To answer this questions, we developed a workflow for the main study:

- All vessels were reviewed using all main criteria. These were treated as equal.
- If unambiguous labeling based on the main criteria was impossible or if the main criteria were not detectable, the additional criteria were applied.

## Main Study

### Subjects

In the main study, 24 eyes from 12 healthy volunteers with 462 labeled vessels were investigated by two independent raters (JM, CE) (7 males, mean age 41.25±13.23).

The inclusion criteria for the subjects were: normal visual acuity (20/20), normal visual field and no ocular, metabolic or neurological diseases.

### Methods

cSLO IR-Images were obtained and labeled as in the exploratory part of the study. All volunteers were examined by an ophthalmologist (FA). CFP imaging was performed using a 30° lens focused on the macula (Visucam, Carl Zeiss Meditech, Berlin, Germany). Photographs were viewed in the Zeiss Visupac 4.2. software (Carl Zeiss Meditech, Berlin, Germany). The vessels in these fundus images were labeled by the ophtalmologist and used as reference for the labeling of the cSLO IR images. Hand-labeling is an established method for finding a baseline [24].

The two cSLO-raters were blinded regarding the results of the CFP.

The criteria were used in the sequence indicated above until unequivocal labeling of the vessels became possible, starting with the main criteria and then using additional criteria, if needed. The number of criteria we applied ranged between one and eight. Each vessel was evaluated applying all main criteria supposing an equal status between the main criteria.

All steps of labeling were documented.

Moreover, using a two point system, we rated the image quality of all vessels as excellent (two points), medium (one point) or insufficient (zero points).

- picture sharpness: 1 point
- identifiability of the lumen of the vessel: 1 point

These ratings served to roughly reflect the cSLO picture quality.

Cohen's kappa was used to measure “inter-rater agreement” between the two raters to assess test reliability.

In the next step, we compared labeled cSLO IR-images and labeled CFP. If the two raters' results were identical, we compared them to the ophtalmologist's as reference. Analysis was performed by a fourfold table, chi-squared test and calculation of kappas to obtain a “test reference agreement”. The test-reference agreement describes the agreement between our new test and the ophthalmologist's as reference. If the two raters (CE, JM) results were not identical, they were declared as a disagreement.

Cohen's kappa was used to measure a test reference agreement between the result of the test and the ophthalmologist's. Additionally, Pearsons's chi-squared test and Fisher's exact test were used to recognize potential correlations.

### Results

#### Inter-rater testing

In the first step, the reliability of our test was determined. The test reached a kappa of κ = 0.840 by labeling 462 vessels. In our control sample, the disagreement-rate between the test and the ophthalmologist's results was 8%. In 1.5% of the cases, vessels were labeled as unknown.

Inter-rater agreement in the main study was better than inter-rater agreement in the exploratory study (Kappa exploratory study: 0.602).

Our exploratory study revealed the difficulties involved in labeling smaller vessels in particular, since the three main criteria often were not applicable for these vessels.

To analyze the quality and the newly established hierarchy of the revised criteria, all vessels were subdivided into two groups to which the following criteria apply:

- 1^st^ choice: The vessel is labeled based on two or three main criteria (MC). Complementary use of one to five additional criteria (AC) is possible, but not mandatory. (≥ 2MC + X AC)
- 2^nd^ choice: The vessel is labeled based on one or less (zero) main criteria. Complementary use of one to five additional criteria is possible, but not mandatory. (≤ 1MC + X AC)

The above classification was selected based on the assumption that the main criteria were the most important ones (heuristic method).

The first and second groups were compared in a fourfold table (table 1).

**Table 1:** Inter-rater-agreement for 1^st^ and 2^nd^ choice.

inter-rater-agreement of the OCT-raters					total
			no agreement	agreement	
	1st choice	absolute	3	257	260
		relative	1.2%	98.8%	100%
	2st choice	absolute	34	168	202
		relative	16.8%	83.2%	100%
total			37	425	462
			8%	92%	100%

Pearson's Chi-squared test and Fisher's exact test revealed a highly significant (p<0.001) difference between the groups and inter-rater agreement.

The Chi-squared test revealed a very high correlation between the use of first-choice criteria and inter-rater agreement. Also, we were able to demonstrate that application of second-choice criteria correlates with poorer inter-rater agreement.

To clarify this observation, kappa coefficients were calculated for the first- and second-choice groups separately:

Inter-rater agreement first choice: κ =  0.976

Inter-rater agreement second choice: κ =  0.673

Further analysis of the first choice group revealed the importance of the additional criteria:

- In 50%, the blood vessel was labeled based on two main criteria plus additional criteria.
- In 15%, the blood vessel was labeled based on three main criteria plus additional criteria.

All in all, 65% of the vessels labeled based on the first-choice approach were labeled using additional criteria for support, therefore they play a crucial role for the analysis.

#### Validity of the test

The second step was to determine the validity of the test. The reference ophthalmologist labeled 85.8% (387 of 462) of the vessels as determinable. For the 387 vessels, Cohen's kappa between the ophthalmologist's result and the results of the cSLO raters was κ = 0.803.

The correlation respectively the values of kappa between the cSLO raters and the reference are the measure for the correctness of the test. The contingency table comparing the test and reference results is presented in table 2. Moreover, test sensitivity and specificity were calculated (table 3).

**Table 2:** Test-reference agreement of 387 identifiable vessels.

test-reference agreement		reference labeled		total
		artery	vein	
**test labeled**	artery	190	31	221
		86%	14%	100%
	vein	7	159	166
		4.2%	95.8%	100%
total		197	190	387
		50.9%	49.1%	100%

**Table 3:** Sensitivity, specificity and positive predictive value of the test.

test-sensitivity for arteries		0.964	(190/197)
test-sensitivity for veins		0.837	(159/190)
test-sensitivity for all vessels		0.902	(349/387)
test-specificity for arteries		0.837	
test-specificity for veins		0.964	
positive predictive value for arteries		0.860	(190/221)
positive predictive value for veins		0.958	(159/166)

The cases were again divided into the first- and second-choice groups. Pearson's Chi-squared test and Fisher's exact test yielded a highly significant (p<0.001) difference between the first- and second-choice groups and the correct labeling result (table 4).

**Table 4:** Connection between the test results and the 1^st^ and 2^nd^ choice.

		Result			total
		incorrect	correct	unidenti-fiable	
1^st^ choice	observed frequency	5	252	3	260
	expected frequency	21.4	196.4	42.2	260
	relative	1.9%	96.9%	1.2%	100%
2^nd^ choice	observed frequency	33	97	72	202
	expected frequency	16.6	152.6	32.8	202
	relative	16.3%	48.1%	35.6%	100%
total	observed frequency	38	349	75	462
	expected frequency	38	349	75	462
	relative	8.2%	75.6%	16.2%	100%

The first choice group included 257 (66%), and the second-choice group 130 of the identifiable blood vessels. Kappa values were calculated both for the first- and second-choice groups:

For the first-choice group, the test-reference agreement was κ = 0.960, and for the second-choice group κ = 0.506.

96% of the undeterminable vessels and 87% of the incorrectly labeled vessels were found in the second-choice group. This distribution also revealed a very highly significant difference (p<0.001) between the groups.

#### Distribution of the criteria applied

To identify the criteria which correlated with incorrect results, we analyzed the frequency distribution of the results (table 5).

**Table 5:** Correlation between main criteria and test results.

case	number of main criteria used	result			total
		incorrect	correct	unidentifiable	
2^nd^ choice	0	27	58	62	147
	1	6	39	10	55
1^st^ choice	2	5	127	3	135
	3	0	125	0	125
		38	349	75	462

The result shows:

- In those cases where all three main criteria had been applied, all blood vessels were labeled correctly (0% incorrect or unidentifiable).
- If two main criteria were used, 6% of the vessels were labeled incorrectly or unidentifiable (8/135).
- Using one main criterion brought a colorful picture of results. In this case, 30% of the blood vessels were incorrectly labeled or unidentifiable (16/55).
- The use of zero main criteria resulted in:
  - ○ a very high rate of unidentifiable vessels (42%; 62/147), and
  - ○ a high rate of incorrectly labeled vessels (18%; 27/147).

Secondly, the **second-choice** cases were split into two groups (see column “cases” in table 5):

- the group **using one main criterion** to analyze the questions:
  - ○ Are one or more of the additional criteria responsible for the wrong results?
  - ○ Are one or more of the additional criteria responsible for the right results?
- the group **using zero main criteria** to answer three questions:
  - ○ Did one or more criterion have a falsifying effect?
  - ○ Is the high rate of undeterminable vessels an indication of poor illustration quality?
  - ○ Does labeling vessels without using any main criteria make sense?

The kappa values for the split second-choice group were calculated separately. If one main criterion and arbitrary additional criteria were used, the test-reference agreement was κ =  0.712. If zero main criteria and arbitrary additional criteria were used, the test-reference agreement was κ =  0.361.

Remember: The test-reference agreement of the second choice group as a whole amounted to: κ =  0.506. In table 6, the frequency of the additional criteria in the different cases was analyzed:

**Table 6:** Distribution of additional criteria, expected frequency means all AC are on an equal level, p-value for the difference between expected and observed frequency.

	criterion	relative frequency	observed frequency	expected frequency	p-value
all identifiable vessels (387)	AC_3	30.2%	117		0.0001
	AC_2	16.0%	62		0.0790
	AC_1	15.0%	58	50.8	0.2588
	AC_4	4.1%	16		0.0001
	AC_5	0.3%	1		0.0001
first choice (260)	AC_3	20%	52		0.0001
	AC_2	11.2%	29		0.0014
	AC_1	1.5%	4	17.2	0.0004
	AC_4	0.4%	1		0.0001
	AC_5	0%	0		0.0001
second choice (202)	AC_1	53%	107		0.0001
	AC_3	41%	83		0.0001
	AC_2	18.8%	38	50.0	0.0578
	AC_4	9.9%	20		0.0001
	AC_5	1%	2		0.0001
correctly labeled vessels (349)	AC_3	29.5%	103		0.0001
	AC_2	16.9%	59		0.0026
	AC_1	11.5%	40	41.6	0.7814
	AC_4	1.4%	5		0.0001
	AC_5	0.3%	1		0.0001

The overview shows that three of the five additional criteria were used very often (AC_1, AC_2, AC_3). The other two additional criteria were used significantly rarer [25]. Unlike in all other cases, AC_1 was the most often used criterion for the second-choice group. The frequencies of the three main criteria were calculated in the same way and did not reveal any significant differences in their distribution (table 7).

**Table 7:** Distribution of main criteria, expected frequency means all MC are on an equal level, p-value for the difference between expected and observed frequency.

	criterion	relative frequency	observed frequency	expected frequency	p-value
all identifiable vessels (387)	MC_1	60.5%	234		0.6264
	MC_2	59.4%	230	228	0.8712
	MC_3	56.8%	220		0.5164
first choice (260)	MC_1	86%	224		0.4522
	MC_2	86.9%	226	215	0.3582
	MC_3	75%	195		0.0948
second choice (202)	MC_1	6%	12		0.0700
	MC_2	4%	8	18	0.0032
	MC_3	17.3%	35		0.0001
correctly labeled vessels (349)	MC_1	66.5%	232		0.4436
	MC_2	64.8%	226	223	0.7844
	MC_3	60.2%	210		0.2986

#### Analysis of the criteria used in the second-choice case

For the second-choice group, the rate of correct results showed no significant difference between using one or more additional criterion (p = 0.08), implying that more additional criteria did not improve the test result. Regarding the quality of the different additional criteria, we found a highly significant correlation between the number of correct results and the additional criterion used for the second-choice group (tables 8 and 9). Pearson's Chi-squared test and Fisher's exact test showed a highly significant (p<0.001) difference between the additional criterion applied and the test result for using one main criterion (table 8). This significant difference was also verifiable in case of using zero main criteria (table 9).

**Table 8:** Correlation between application of additional criteria and test result based on one main criterion.

Using one main criterion in the second-choice group		result		total
		correct	incorrect or unidentifiable	
AC_1	observed frequency	3	5	8
	expected frequency	5.6	2.4	8
	relative	37.5%	62.5%	100%
AC_2	observed frequency	13	1	14
	expected frequency	9.8	4.2	14
	relative	92.9%	7.1%	100%
AC_3	observed frequency	31	9	40
	expected frequency	28.1	11.9	40
	relative	77.5%	22.5%	100%
AC_4	observed frequency	0	5	5
	expected frequency	3.5	1.5	5
	relative	0.0%	100%	100%

**Table 9:** Correlation between application of additional criteria and test results based on zero main criteria.

Using zero main criteria in the second-choice group		Result		total
		correct	incorrect or unidentifiable	
AC_1	observed frequency	33	66	99
	expected frequency	42.7	56.3	99
	relative	33.3%	66.7%	100%
AC_2	observed frequency	17	7	24
	expected frequency	10.4	13.6	24
	relative	70.8%	29.2%	100%
AC_3	observed frequency	24	19	43
	expected frequency	18.6	24.4	43
	relative	55.8%	44.2%	100%
AC_4	observed frequency	4	11	15
	expected frequency	6.5	8.5	15
	relative	26.7%	73.3%	100%
AC_5	observed frequency	1	1	2
	expected frequency	0.9	1.1	2
	relative	50%	50%	100%

Tables 6, 8 and 9 show that the use of AC_1 correlated with a high rate of wrong results. AC_4 and AC_5 were sparely used and are statistically not evaluable, and the use of AC_2 and AC_3 correlated with correct results.

The kappas for application of one main criterion and zero main criteria were calculated excluding the use of the criteria AC_1, AC_4 and AC_5:

one main criterion: κ = 0.940

zero main criteria: κ = 0.529

Also, the test-reference agreement of the whole second-choice group without these criteria was calculated: κ = 0.745. The consequences of eliminating AC_1, AC_4 and AC_5 are summarized in table 10.

**Table 10:** Test reference agreement of the second-choice group before and after elimination of AC_1, AC_4 and AC_5.

	before elimination of AC 1,4,5	after elimination of AC 1,4,5
whole second choice group	κ = 0.506	κ = 0.745
using one main criterion	κ = 0.712	κ = 0.940
using zero main criteria	κ = 0.361	κ = 0.529

Because of the high frequency of AC_1 in the case of zero main criteria (table 9; AC_1 was used in 99/183 (54%) of cases), this additional criterion was considered separately. By using only AC_1, (while eliminating all other criteria), the test-reference agreement was κ = 0.291.

## Discussion

Our study aimed to establish valid and reliable criteria for blood vessel labeling in cSLO IR-images, obtained by SD-OCT scanners used in parallel to OCT images. In doing so, we compared eight criteria extracted from the ophthalmological literature.

We set up a hierarchy of these criteria with three main and five additional criteria.

*Main criteria*:

1. *The central light reflex is wider in arteries and smaller in veins.*
2. *Arteries are brighter than veins.*
3. *Arteries are thinner than veins.*

*Additional criteria*:

1. *Arteries and veins alternate near the optic disc.*
2. *Arteries never cross arteries and veins never cross veins.*
3. *Vessels should be seen in the context of the vessel tree.*
4. *Arteries take a straighter course than veins.*
5. *Angles between crossing blood vessels are almost 90°, whereas angles between outgoing vessels are between 30° and 45°.*

Moreover, blood vessels which were labeled applying two or more main criteria yielded better test results than vessels labeled based on less than two main criteria. These two cases were analyzed as first and second choice.

For the cases ≥2MC + X AC (first choice), an almost perfect inter-rater agreement (κ = 0.976), an almost perfect correctness rate (κ = 0.960) and a very low rate of unidentifiable vessels (1.15% (3/260)) was shown.

We demonstrated that the three main criteria were equally important and equally often used.

In case of using additional criteria we found, that for all correctly labeled vessels of the first-choice group, AC_2 and AC_3 led to correct results (table 6). However, only 66% of all potentially identifiable vessels were covered by the first choice. Consequently, using first choice only would leave one third of the vessels undetermined.

The second-choice group (≤1MC + X AC) presented a different picture of the test results and criteria frequencies. In an attempt to find a reason for the insufficient test results for the second-choice group, we subdivided this group into two subgroups, one using one main criterion and the other using zero main criteria.

It turned out that AC_2 and AC_3 were the only additional criteria which yielded good results in the second choice cases. Elimination of the other additional criteria caused the test-reference agreement of the second choice cases to increase significantly.

### The test in its entirety

With the exception of the second-choice group, the additional criterion AC_3 was the most frequently used one (table 6) in all cases. Furthermore, analysis of frequencies suggests that AC_4 and AC_5 might be irrelevant for first and second choice (whole test) (table 6).

**Figure 7:**
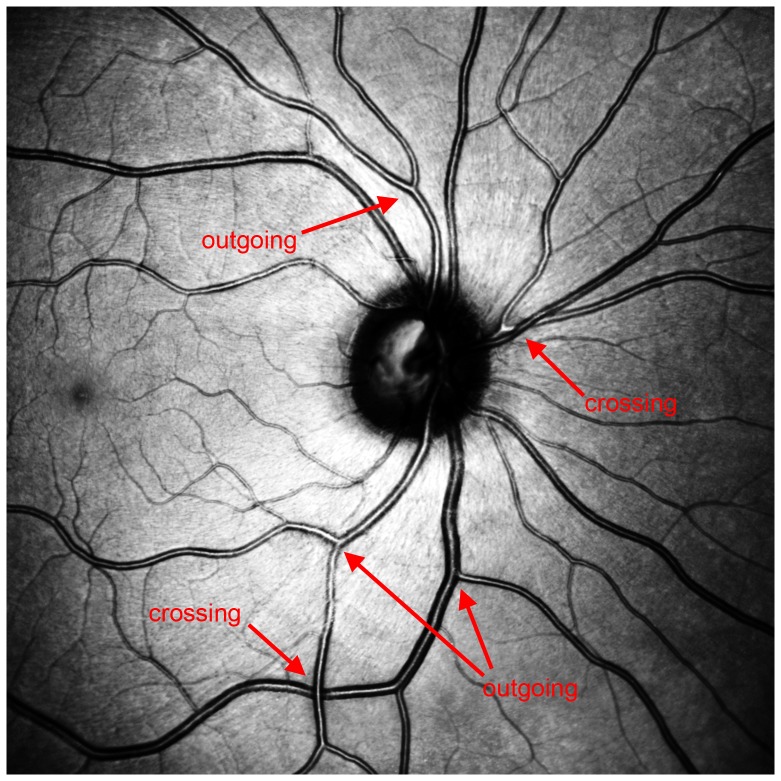
Both the crossing and the outgoing vessels show 90° and 30°-45° angles.

In contrast to [16], we were able to demonstrate both statistically and empirically that criterion AC_5 is incorrect. To give an example, figure 7 shows blood vessels crossing at an angle of 45°, and blood vessels branching at a 90° angle. For the new test, AC_1, AC_4 and AC_5 were eliminated.

There are two different options for labeling:

1. The test should label all vessels based on the main criteria; if no main criterion can be detected or if non-ambiguous labeling is not possible, use of AC_2 and/or AC_3 is allowed.
2. The test should label only those vessels to which one or more main criteria apply. If no main criterion is detectable, the vessel should be classified as unidentifiable.

In connection with the first option, 18% (n = 69) of the potentially identifiable blood vessels remained undetected. The kappa of the test-reference agreement was at κ =  0.916. For the second option, 25% (n = 98) of the potentially identifiable vessels were not detected by the test. The kappa of test-reference agreement was κ =  0.957.

The measured image quality of the missed blood vessels is shown in table 11.

**Table 11:** Quality of potentially identifiable missed vessels applying the 1^st^ or 2^nd^ test option.

Quality of missed vessels	1^st^ option	2^nd^ option
good	8.7% (6)	6.1% (6)
medium	24.6% (17)	25.5% (25)
bad	66.7% (46)	68.4% (67)

Consequently, in the context of the first option, 91.1% of the missed vessels and 93.9% for the second option do not have a good quality. The quality of the detected vessels in the first possibility was good in 64%, in the second possibility in 70% of the cases.

A possible explanation for the fact that vessels are not identifiable is the different imaging technique of CFP and cSLO. The OCT scanner produces black-and-white cSLO IR images; the fundus image on the other hand is a colored photograph. A colored picture contains more information about the vessels, in particular on blood oxygenation.

## Conclusion

Two new test forms with excellent results are possible:

In the first version, the test labels all vessels by the main criteria, and if no main criterion can be detected or if non-ambiguous labeling is not possible, using additionally criteria (AC_2 and/or AC_3) is allowed.

In the second version, only those vessels in the test to which one or more main criteria apply are subjected to labeling. If no main criterion can be detected, the vessel should be classified as unidentifiable.

The first version yields a higher rate of identifiable vessels, the second version a higher rate of security in labeling. In the first version, the kappa of κ = 0.916 remains almost perfect. So, to include as many vessels as possible, we prefer the first version of the test. This benefit outweighs the lower level of accuracy.

**Figure 8:**
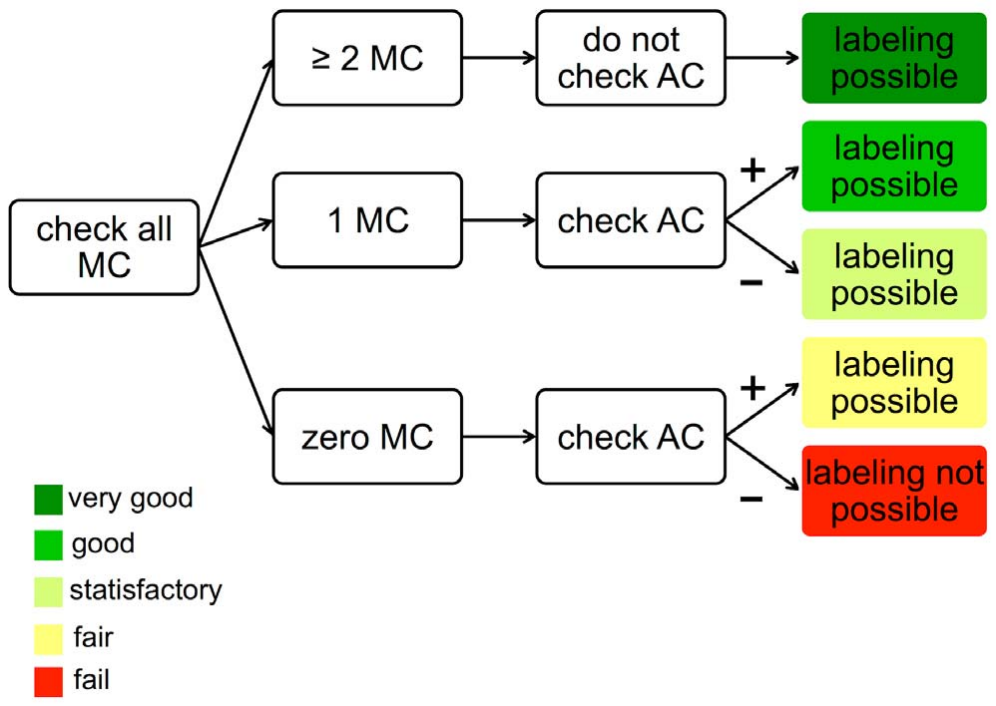
Workflow for correct vessel labelling in cSLO images. The different levels of test security are visualized by five colours. MC  =  main criteria: The central light reflex is wider in arteries and smaller in veins. Arteries are brighter than veins. Arteries are thinner than veins. AC  =  additional criteria: Arteries never cross arteries and veins never cross veins. Vessels should be seen in the context of the vessel tree.

Ultimately, the user of the test has to define an objective before starting the test. This objective will depend on what the data will be used for. Before the labeling is run, the user will define whether the goal is a high rate of identifiable vessels or maximum test-accuracy. Figure 8 shows a hands-on workflow for vessel labeling and visualizes the different levels of test security.

The disadvantage of our method is that image resolution here does not attain the level reached via CFP. Moreover, cSLO-images are black-and-white shots only, so the level of information is technically limited. Moreover, the test did not determine all vessels which could be identified in the reference.

In spite of these curtailments, the method presented here, which involves using cSLO IR-images produced by an OCT scanner for the purpose of investigating blood vessels has many benefits:

high reliability (κ = 0.840)
high validity (κ = 0.957)
time-saving method – only one rater required.

**Figure 9:**
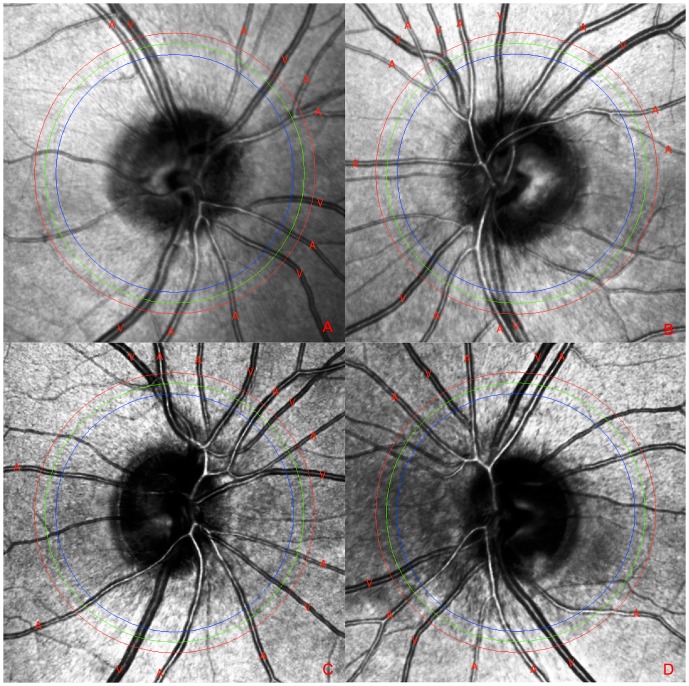
As an example for vascular diseases cSLO images of four eyes of two patients are shown. Picture A and B belong to a patient suffering from cerebral vasculitis. Picture C and D arise from a patient who is affected by giant-cell arteriitis. A change of vessel characteristics is not noticeable. Vessel labeling was performed using the new workflow.

The test was developed based on data from healthy subjects. This fact brings a few restrictions. In course of ethical reasons it was not possible to use fluorescein angiography in place of CFP as ophthalmologic reference. Moreover the small number of subjects in the study precludes a definite evidence. To get an impression of the test results in pathologic entities the workflow was tested additionally on four eyes of two patients. One was suffering from cerebral vasculitis, the other one was affected by giant-cell arteriitis. Diagnosis was made by cerebral MRI and vessel biopsy. Even in these pathological conditions it was possible to label the vessels using the developed workflow. The cSLO images of them are shown in figure 9. The characteristics of arteries and veins in the images of these sick two patients do not differ from healthy subjects. Thus, the workflow for vessel labeling could be applied exemplary in eyes with vascular diseases as well. To detect differences in the vessel morphology of sick and healthy subjects measurements, e.g. of the vessels' diameter, in cSLO images are necessary. This could be done in future studies. Recently, the workflow was also successfully used for vessel labeling in CADASIL patients [Alten et al., manuscript submitted].

The examination of vessels in cSLO images offers a straightforward, practicable extension of the application of OCT technique into neurology without tying up further technical resources. Blood vessel examination and screening has a high clinical potential. Spectral domain optical coherence tomography using automated eye tracking and image alignment based on cSLO images is very fast, non-invasive and little personel intensive and combines several aspects of retinal examination in a single device.

Many clinical applications are conceivable; in particular vascular neurological diseases like cerebral vasculitis, CADASIL or cerebral micro-/macroangiopathies might be revealed in abnormal vessels in OCT and cSLO-images.

But since widespread diseases like dementia or stroke also have been shown to correlate with retinal vessel changes in CFP, SD-OCT technology has the potential to open an even wider field of medical applications as it combines knowledge on cerebral and retinal diseases in one application. The increasing use of SD-OCT imaging, originally used in ophthalmology, shows the increasing overlap between the different medical disciplines.
